# Antemortem diagnosis of pulmonary tumor thrombotic microangiopathy in a patient with recurrent breast cancer: a case report

**DOI:** 10.1186/s12885-016-2721-3

**Published:** 2016-08-22

**Authors:** Yui Takahashi, Hironori Uruga, Takeshi Fujii, Sayaka Mochizuki, Shigeo Hanada, Hisashi Takaya, Atsushi Miyamoto, Nasa Morokawa, Atsuko Kurosaki, Kazuma Kishi

**Affiliations:** 1 Department of Respiratory Medicine, Respiratory Center, Toranomon Hspital, 2-2-2 Toranomon, Minato-ku, Tokyo, 105-8470 Japan; 2 Department of Pathology, Toranomon Hospital, 2-2-2 Toranomon, Minato-ku, Tokyo, 105-8470 Japan; 3 Department of Diagnostic Radiology, Fukujuji Hospital, 3-1-24 Matsuyama, Kiyose-shi, Tokyo, 204-8522 Japan; 4 Okinaka Memorial Institute for Medical Research, Tokyo, Japan

**Keywords:** Pulmonary tumor thrombotic microangiopathy, Breast cancer, Tumor embolism, Trastuzumab, Human epidermal growth factor receptor 2

## Abstract

**Background:**

Pulmonary tumor thrombotic microangiopathy (PTTM), a rare complication of advanced cancer, is histologically characterized by tumor embolisms and fibrocellular intimal proliferation of small pulmonary arteries and arterioles. PTTM usually has an extremely poor prognosis, and antemortem diagnosis is very difficult.

**Case presentation:**

A 65-year-old woman with a 5-year history of clinical stage IIA (T2N0M0) invasive ductal carcinoma of the left breast was hospitalized for worsening shortness of breath, hemoptysis, and cough since 2 months. She had previously received neoadjuvant chemotherapy and left mastectomy. Because the cancer cells were positive for human epidermal growth factor receptor 2 (HER2), four cycles of trastuzumab had been administered as adjuvant chemotherapy. On admission, chest computed tomography (CT) showed peripheral consolidations in both the lower lobes and a mediastinal mass. Specimens obtained on video-assisted thoracoscopic surgical biopsy revealed tumor cell embolism, intimal fibrocellular proliferation of small arteries, fibrin thrombi, recanalization, and infarction in the left lower lobe, as well as metastasis to the mediastinal pleura. Immunohistochemical staining of the tumor cells revealed positivity for HER2, and a diagnosis of recurrent breast cancer with PTTM was made. Four cycles of trastuzumab resulted in rapid improvement of her symptoms and CT findings of peripheral consolidations and the mediastinal mass.

**Conclusion:**

An antemortem diagnosis of PTTM was made in a patient with HER2-positive recurrent breast cancer. Trastuzumab was effective for not only breast cancer but also PTTM.

## Background

Pulmonary tumor thrombotic microangiopathy (PTTM) is a rare complication of advanced cancer; it is histologically characterized by tumor embolisms and fibrocellular intimal proliferation of small pulmonary arteries and arterioles [1]. PTTM causes rapidly progressive pulmonary hypertension, right heart failure, and death in a few days. It is usually diagnosed on autopsy and is rarely diagnosed antemortem [2–8]. The most frequent underlying malignancy is gastric adenocarcinoma, while breast cancer is an infrequent cause of PTTM [1, 9, 10]. Herein, we report a patient with human epidermal growth factor receptor 2 (HER2)-positive recurrent breast cancer with accompanying PTTM without pulmonary hypertension; the diagnosis was made antemortem, and the patient responded well to trastuzumab therapy.

## Case presentation

A 65-year-old woman with a 5-year history of clinical stage IIA (T2N0M0) invasive ductal carcinoma of the left breast was hospitalized for worsening shortness of breath, hemoptysis, and cough since 2 months. Her breast carcinoma was 32 mm in diameter at diagnosis, with a histologic grade of 3 and nuclear grade of 3. Immunostaining for the estrogen receptor and progesterone receptor revealed negativity for both, but the tumor cells were positive for HER2. The patient had received neoadjuvant chemotherapy with four cycles of epirubicin and cyclophosphamide, followed by four cycles of trastuzumab and paclitaxel. A left mastectomy had been performed, and the surgical specimen showed no residual cancer. After the operation, four cycles of trastuzumab had been administered as adjuvant chemotherapy; however, trastuzumab had been discontinued because of heart failure. Echocardiography showed diffuse and moderate impairment of left ventricular contraction and a decrease in ejection fraction from 65.8 % to 36 %. Seven months after stopping trastuzumab administration, a repeat echocardiography revealed that her ejection fraction had recovered. Since then, she had been followed-up without treatment for breast carcinoma until this readmission.

**Fig. 1:**
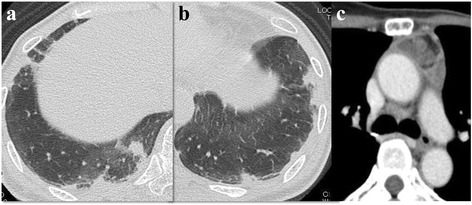
Chest computed tomography scans on admission. **a**, **b** Irregular shaped peripheral consolidations in both the lower lobes. **c** A heterogeneously enhanced mediastinal mass

**Fig. 2:**
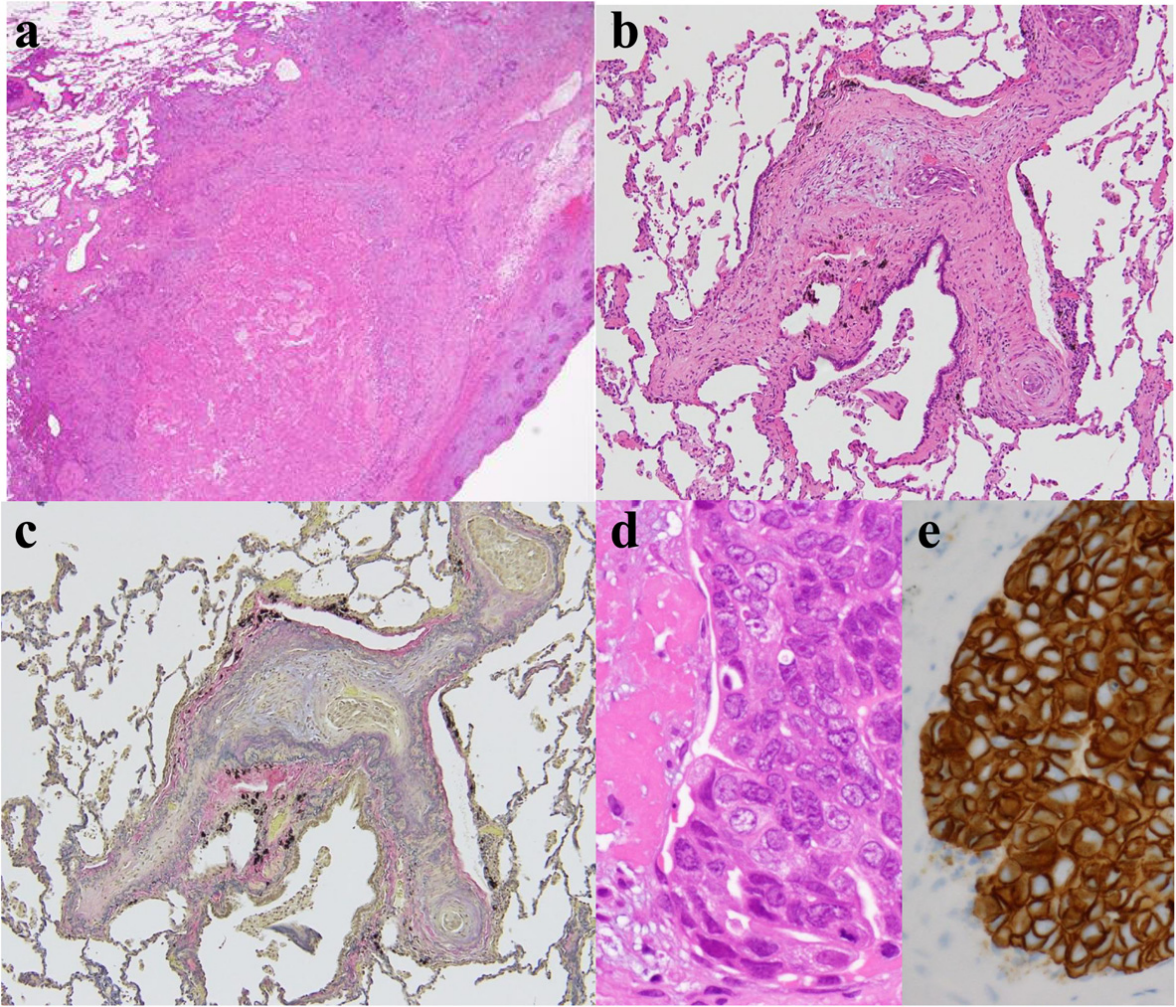
Histopathological findings of the surgical specimens. **a** Widespread infarction (hematoxylin and eosin staining, ×2); **b** Tumor fibrin embolism in a pulmonary arteriole (hematoxylin and eosin staining, ×10); **c** Fibrocellular intimal proliferation (elastic van Gieson staining, ×10); **d** Tumor cells (at increased magnification, hematoxylin and eosin staining, ×60); and **e** Tumor cells demonstrating HER2 positivity (immunohistochemical staining, ×60)

On admission, the patient showed normal auscultation findings. A chest radiograph showed faint infiltrates at the base of both the lungs. Arterial blood gas analysis using room air indicated minimal hypoxemia: pH, 7.42; PaCO_2_, 42 mmHg; and PaO_2_, 78 mmHg. D-dimer levels were slightly increased to 1.2 μg/mL (normal, <1.0 μg/mL). Serum levels of carcinoembryonic antigen and HER2 were elevated to 57.0 μg/L (normal, 0.8–4.8 μg/L) and 64.9 ng/mL (normal, <15.2 ng/mL), respectively. Contrast-enhanced computed tomography (CT) scans revealed peripheral consolidation with ground-glass opacity in both the lower lobes and a heterogeneous mediastinal mass (Fig. 1). ^18^F-fluorodeoxyglucose (FDG)-positron emission tomography demonstrated increased FDG uptake in the peripheral consolidation and the mass. Echocardiography revealed normal contractions without any finding suggestive of pulmonary hypertension. Because transbronchial lung biopsy did not lead to any diagnosis, video-assisted thoracoscopic surgery (VATS) lung biopsy was performed. The surgical specimens of the peripheral area of the left lower lobe and the mediastinal mass revealed tumor cell embolism, intimal fibrocellular proliferation of small arteries, fibrin thrombi, recanalization, and infarction in the left lower lobe, as well as metastasis to the mediastinal pleura (Fig. 2). Immunohistochemical staining of tumor cells revealed positivity for mammaglobin, gross cystic disease fluid protein 15, HER2 (3+), and vascular endothelial growth factor. Accordingly, a diagnosis of recurrent breast cancer with PTTM was made.

**Fig. 3:**
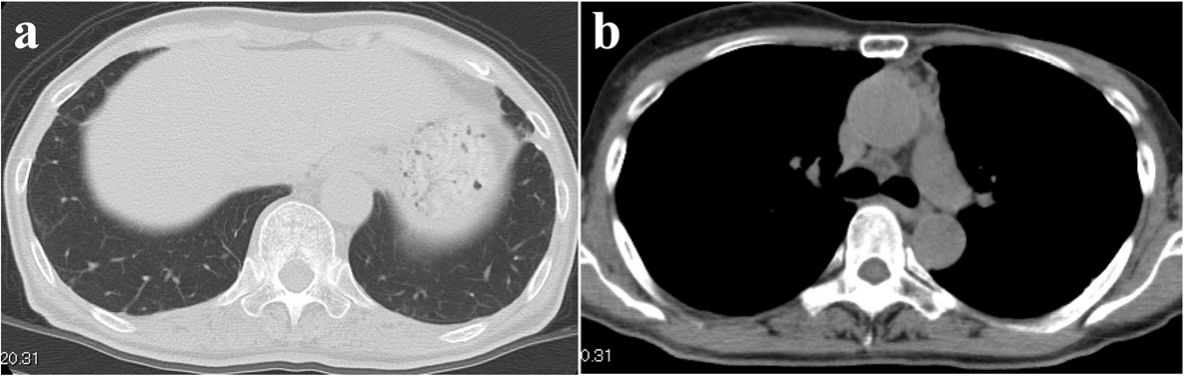
Chest computed tomography scans after trastuzumab therapy showing improvements of (**a**) consolidations in both the lower lobes and (**b**) the mediastinal mass.

Retreatment with trastuzumab therapy rapidly improved the symptoms and CT findings of peripheral consolidation and the mass, resulting in partial remission (Fig. 3). After four cycles of trastuzumab therapy, it was stopped owing to heart failure. The patient visited another hospital and is alive more than 2 years after the diagnosis of PTTM.

## Discussion

We report a patient with recurrent breast cancer to the left lung accompanied by PTTM, which was diagnosed antemortem. The onset was subacute, and respiratory failure was absent. Although transbronchial lung biopsy could not help in making a diagnosis, the results obtained with VATS lung biopsy led to the diagnosis. Treatment with trastuzumab, a humanized monoclonal antibody that targets the extracellular domain of HER2, rapidly resolved the patient’s symptoms and CT findings.

Because PTTM is usually diagnosed postmortem, the clinical manifestations are not fully understood. Unlike in the present patient, the prognosis is known to be extremely poor. The incidence of PTTM is 0.9–3.3 % in autopsy patients with carcinoma, and the most common origin was gastric carcinoma, followed by lung and breast carcinoma [1, 9, 10]. The proposed mechanism of PTTM is that tumor cells activate the coagulation systems and release inflammatory mediators and growth factors including vascular endothelial growth factor, resulting in thrombosis, fibrocellular intimal proliferation, and luminal stenosis [5]. Elevated serum levels of D-dimer and fibrin degradation products have been reported [10]. Patients with PTTM frequently develop severe pulmonary hypertension and disseminated intravascular coagulation, but our patient did not have either.

PTTM differs from tumor emboli in terms of accompanying activation of the coagulation system and intimal proliferation in small arteries [1]. Clinically, patients with PTTM usually show rapid worsening of respiratory function, with or without pulmonary hypertension. Unlike tumor emboli, the proximal pulmonary arteries do not show defects on contrast-enhanced CT scans of patients with PTTM.

In the present patient, a prominent CT finding was the peripheral consolidation in both the lower lobes, which was pathologically correlated with infarction. After breast cancer treatment, chest CT can show abnormalities due to various causes including radiation pneumonitis, chemotherapy-induced interstitial changes, pulmonary infection under immunosuppressed condition, cancer related thromboembolic events including PTTM, and pulmonary metastasis [11]. Among them, CT findings of PTTM include consolidations, ground-glass opacities, small nodules, and tree-in-bud appearance [2, 5, 7, 10, 12]. We should consider breast cancer recurrence with PTTM as a differential diagnosis if a patient presents with respiratory symptoms and abnormalities on chest CT images, after breast cancer treatment.

To our knowledge, only 10 patients have been successfully diagnosed with PTTM antemortem (Table 1) [2–8, 12, 13]. The primary site was the upper gastrointestinal tract (*n* = 4), breasts (*n* = 3), unknown (*n* = 2), and lungs (*n* = 1). The diagnostic methods for PTTM were transbronchial lung biopsy in five patients and VATS in three. Pulmonary hypertension was detected in five patients. All but one patient received treatment with anti-cancer drug and seven patients survived for more than 3 months. Among them, only the present patient received a molecular-targeted drug trastuzumab that acts against known molecular abnormalities. Trastuzumab therapy was effective for not only breast cancer but also PTTM.

**Table 1:** Previously reported antemortem diagnoses of PTTM

Year	Author	Primary site	Diagnostic method of PTTM	CT findings	PH	Treatment	Survival time
2007	Miyano et al. [5]	Stomach	VATS	Multiple tiny nodules	-	S-1 + dexamethasone + warfarin, aspirin	9 months
2008	Noguchi et al. [12]	Stomach	TBLB	Tiny nodules	+	No treatment	10 days
2008	Uruga et al. [8]	Lung	CT guided biopsy	Consolidation, ground glass attenuation	-	Carboplatin, paclitaxel	7 months
2011	Ishiguro et al. [2]	Unknown	TBLB	Mild interlobular septal thickening, ground glass opacities	+	CHOP(cyclophosphamide, doxorubicin, vincristine, prednisolone)	4 months
2011	Ueda et al. [7]	Esophagus	TBLB	Multiple bilateral diffuse interstitial infiltrative shadows	+	Fluorouracil, nedaplatin	9 days
2012	Kayatani et al. [3]	Unknown	VATS	Centrilobular ultrafine granular shadows	-	S-1, cisplatin	15 months
2013	Ogawa et al. [6]	Gastroduodenum	TBLB	A nodular shadow and septal thicken with a slight amount of pleural effusion	+	Bosentan, epoprostenol + imatinib + S-1, 5-fluorouracil	9 months
2013	Kitamura et al. [4]	Breast	TBLB	Ground glass opacities around the bronchovascular bundles	-	Irinotecan + warfarin	3 months
2014	Higo et al. [13]	Breast	Pulmonary wedge aspiration	Mosaic pattern	+	Bosentan, tadalafil + imatinib, bevacizumab + S-1	12 months
2015	Takahashi et al. (present case)	Breast	VATS	Peripheral consolidation in both lower lobes	-	Trastuzumab	32 months

*PTTM* Pulmonary tumor thrombotic microangiopathy, *PH* pulmonary hypertension, *TBLB* transbronchial lung biopsy, *VATS* Video-assisted thoracoscopic surgery, *S-1* tegafur, *gimeracil* oteracil potassium

Although von Herbay et al. first reported PTTM [1], almost all previous reports have been from Japan. Although we do not know the exact reason for this, there are several possibilities. First, the prevalence of gastric cancer is high in Japan. Second, Japanese patients can access hospital care easily owing to a national insurance system. Lastly, teaching hospitals, including ours, understand the importance of autopsy, and we try to perform autopsy as often as possible.

## Conclusions

PTTM should be included in the differential diagnosis when breast cancer patients exhibit progressive respiratory symptoms with lung infiltrations without pulmonary infection or heart failure. When tumor cells are positive for an oncogenic driver such as HER2, genotype-directed therapy seems promising for the treatment of both cancer and PTTM.

## Abbreviations

CT: Computed tomography
FDG: ^18^F-fluorodeoxyglucose
HER2: Human epidermal growth factor receptor 2
PTTM: Pulmonary tumor thrombotic microangiopathy
VATS: Video-assisted thoracoscopic surgery
